# Evolutionary novelty in gravity sensing through horizontal gene transfer and high-order protein assembly

**DOI:** 10.1371/journal.pbio.2004920

**Published:** 2018-04-24

**Authors:** Tu Anh Nguyen, Jamie Greig, Asif Khan, Cara Goh, Gregory Jedd

**Affiliations:** Temasek Life Sciences Laboratory & Department of Biological Sciences, The National University of Singapore, Singapore; Swiss Federal Institute of Technology Lausanne (EPFL), Switzerland

## Abstract

Horizontal gene transfer (HGT) can promote evolutionary adaptation by transforming a species’ relationship to the environment. In most well-understood cases of HGT, acquired and donor functions appear to remain closely related. Thus, the degree to which HGT can lead to evolutionary novelties remains unclear. Mucorales fungi sense gravity through the sedimentation of vacuolar protein crystals. Here, we identify the octahedral crystal matrix protein (OCTIN). Phylogenetic analysis strongly supports acquisition of *octin* by HGT from bacteria. A bacterial OCTIN forms high-order periplasmic oligomers, and inter-molecular disulphide bonds are formed by both fungal and bacterial OCTINs, suggesting that they share elements of a conserved assembly mechanism. However, estimated sedimentation velocities preclude a gravity-sensing function for the bacterial structures. Together, our data suggest that HGT from bacteria into the Mucorales allowed a dramatic increase in assembly scale and emergence of the gravity-sensing function. We conclude that HGT can lead to evolutionary novelties that emerge depending on the physiological and cellular context of protein assembly.

## Author summary

A central question in evolutionary biology is how novel traits arise. Gravitropism—the orientation of growth based on gravity—evolved independently in different groups of plants and fungi. Dense bodies formed within distinct organelles play a key role by sedimenting under the influence of gravity to activate signaling pathways that orient growth. Here we identify the octahedral crystal matrix protein (OCTIN) as the structural protein that makes up sedimenting vacuolar crystals in the fungal order Mucorales (pin molds). OCTIN appears to have originated by horizontal gene transfer (HGT) from bacteria, but the dominance of Brownian motion at bacterial size scales makes an ancestral role in gravity sensing unlikely, indicating that OCTIN evolved its new function after HGT. However, bacterial OCTIN self-assembles in a manner similar to fungal OCTIN. Our data suggest that fungal OCTIN evolved by retaining elements of the original bacterial assembly mechanism, while acquiring new mutations that increase assembly size. Most genes taken up by HGT encode enzymes whose original and acquired function remains closely related; this study by contrast identifies an assembly-based mechanism for the emergence of novelties through HGT. We speculate that these new functions can emerge depending on changes not only in scale, but also in assembly number, shape, and dynamics.

## Introduction

The acquisition of new protein functions through horizontal gene transfer (HGT) is known to confer selective advantages and enable the occupation of new ecological niches [1–6]. Examples include the acquisition of antibiotic resistance [7], virulence-promoting factors [8], expanded enzymatic capability [9–17], and tolerance of environmental extremes [18,19]. In well-understood cases of HGT, the transferred genes generally encode enzymes whose functions appear to be retained in the recipients.

The ability to sense gravity allows plants and fungi to orient the growth of shoots and roots, and fruiting bodies, respectively. This response, known as gravitropism, depends on sedimentation of dense cytoplasmic bodies [20–22], which generate cell elongation-promoting signals at the cell cortex. Plant gravity sensing is mediated by starch bodies that form within specialized plastids [20]. In the fungi, gravitropism has been demonstrated in the multicellular Basidiomycota [21] and the Mucorales [22]. However, gravity-sensing organelles have only been examined in the Mucoralean *Phycomyces blakesleeanus* [23] where giant single-celled sporangiophores exhibit gravitropism through a combination of buoyant lipid globules and sedimenting protein crystals that form within vacuoles [24]. A crystal-less mutant grows normally, but displays defective gravitropism, indicating that the crystals indeed serve as gravity sensors [24–26]. Similar structures have been observed in other members of the Mucorales [22], suggesting that this function arose early in this lineage. However, its basis and evolutionary origin remain unknown.

Here, we identify the octahedral crystal matrix protein (OCTIN). Phylogenetic analyses indicate that *octin* was acquired from a gram-negative bacterium. Both *Phycomyces* crystals and bacterial OCTIN form disulfide-bonded high-order oligomers, suggesting that they share elements of a conserved assembly mechanism. Given the size of bacterial cells, thermal fluctuations are expected to dominate the movement of OCTIN oligomers. This precludes any speculated role in bacterial gravity sensing. We conclude that HGT of a bacterial *octin* into the common ancestor of the Mucorales is likely to have relieved constraints on OCTIN oligomer size, allowing evolution of the gravity-sensing function. The data exemplify a general mechanism for the evolution of adaptations based on HGT and high-order protein assembly.

## Results and discussion

**Fig 1:**
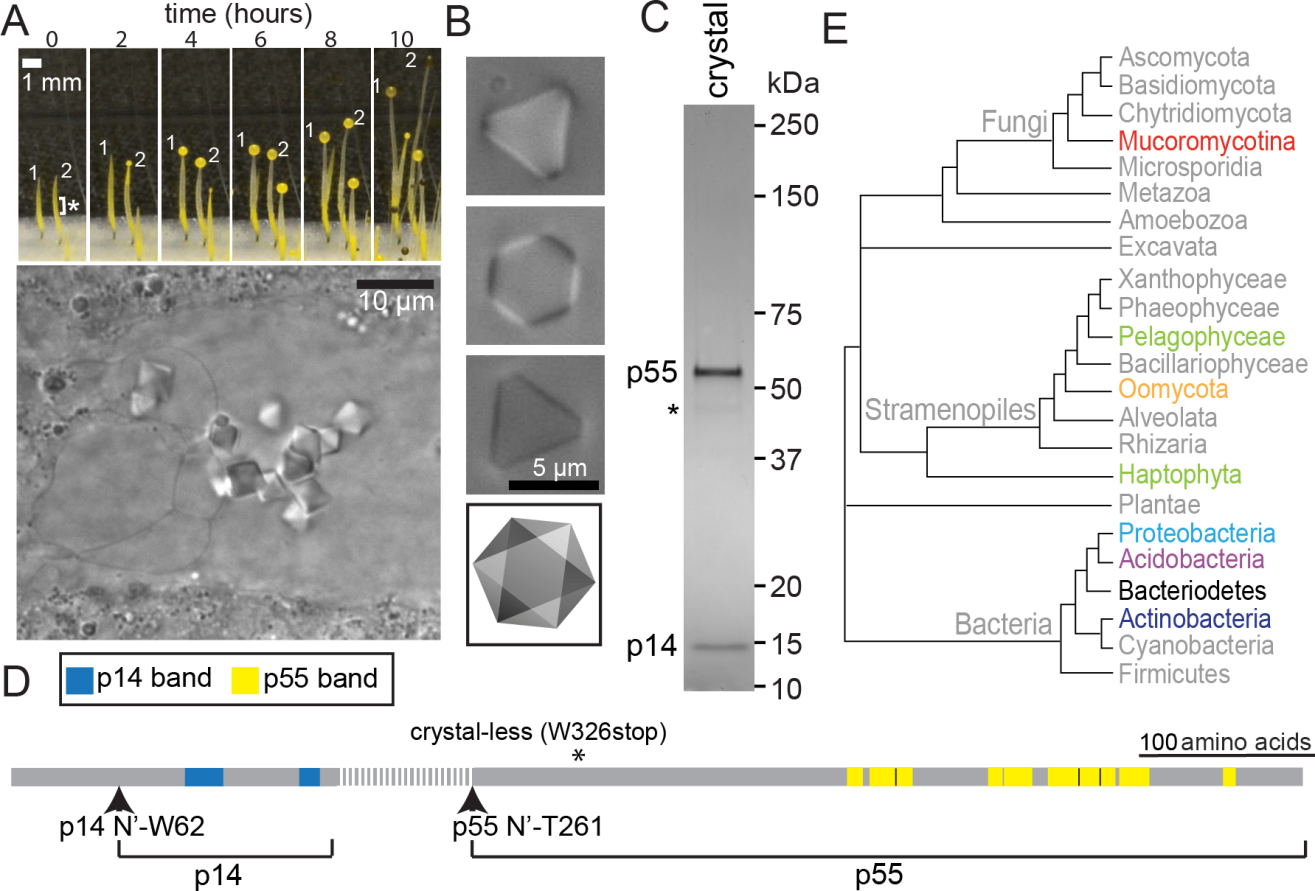
*P*. *blakesleeanus* OCTIN crystals. (A) The upper panel shows *Phycomyces* asexual fruiting body development tracked over the course of 10 hours. The stalk is a single-celled sporangiophore and the sphere at its tip contains nuclei that develop into asexual spores. The asterisk indicates the approximate region where protein crystals occur. The lower panel shows a close-up view of protein crystals within the sporangiophore central vacuole. (B) Three focal planes reveal the octahedral structure of a purified crystal. The bottom panel shows a cartoon of the crystal geometry. The lightest triangular face corresponds to the first panel. The darkest triangular face corresponds to the third panel. (C) The crystal-enriched fraction analyzed by SDS-PAGE. Two prominent proteins, p55 and p14 are indicated. The asterisk identifies a 46-kDa band whose peptides are mapped to the same region as p55 by mass spectrometry. (D) The cartoon depicts the full-length OCTIN sequence. Peptides identified from p14 and p55 are shown in blue and yellow, respectively. The N-termini of the mature proteins are indicated (arrowheads). The dashed line identifies the predicted region removed through proteolytic processing based on the molecular weight of p14. An asterisk marks the position of a stop-codon in the crystal-less mutant. (E) An organismal phylogeny showing the distribution of taxa where full-length OCTIN homologs are found. Names of these taxa are in colored or black labels. Gray colored groups do not contain OCTIN. OCTIN, octahedral crystal matrix protein.

To determine the molecular basis of gravity sensing, we purified vacuolar crystals from *P*. *blakesleeanus* sporangiophores using the method of Ootaki and Wolken (Fig 1) [27]. As previously observed, a highly purified crystal fraction contains two major proteins, p55 and p14 (Fig 1C) [28]. Mass spectrometry indicates that peptides from these bands are derived from the N- (p14) and C-terminus (p55) of a single predicted protein, which we named OCTIN (Fig 1D). Edman degradation defines the N’-termini of p14 and p55 and full-length *octin* transcript is detected exclusively in sporangiophores (Figs 1D and S1A). These data indicate that p14 and p55 are derived through proteolytic processing of an OCTIN precursor. Furthermore, sequencing the *octin* gene from the crystal-less mutant reveals a stop codon at W326 (Figs 1D and S1B). Together, these observations identify two OCTIN-derived proteins as primary components of *Phycomyces* gravity-sensing crystals.

Full-length OCTIN is sporadically present in eukaryotes and bacteria (Figs 1E and S2). In the fungi, OCTIN is found exclusively in members of the Mucoromycotina, suggesting that it was acquired early on in this lineage. Homologs are also found in the protozoan Stramenopiles, including all sequenced Oomycetes, the Pelagophyceae diatom *Aureococcus anophagefferens* and both sequenced Haptophytes (the brown alga *Emiliania huxleyi* and the phytoplankton *Chrysochromulina*). OCTIN also occurs sporadically in diverse bacterial clades, where it is found in Proteobacteria, Acidobacteria, Actinobacteria, and Bacteroidetes (Fig 1E). Mucorales *octin* sequences do not encode a predicted signal sequence, suggesting localization through the cytoplasm-to-vacuole targeting pathway, which has been associated with the import of oligomeric vacuolar resident proteins [29]. Predicted signal sequences are found in OCTIN homologs from gram-negative bacteria and the Oomycetes, suggesting that these proteins are directed to the periplasm and secretory pathway, respectively.

**Fig 2:**
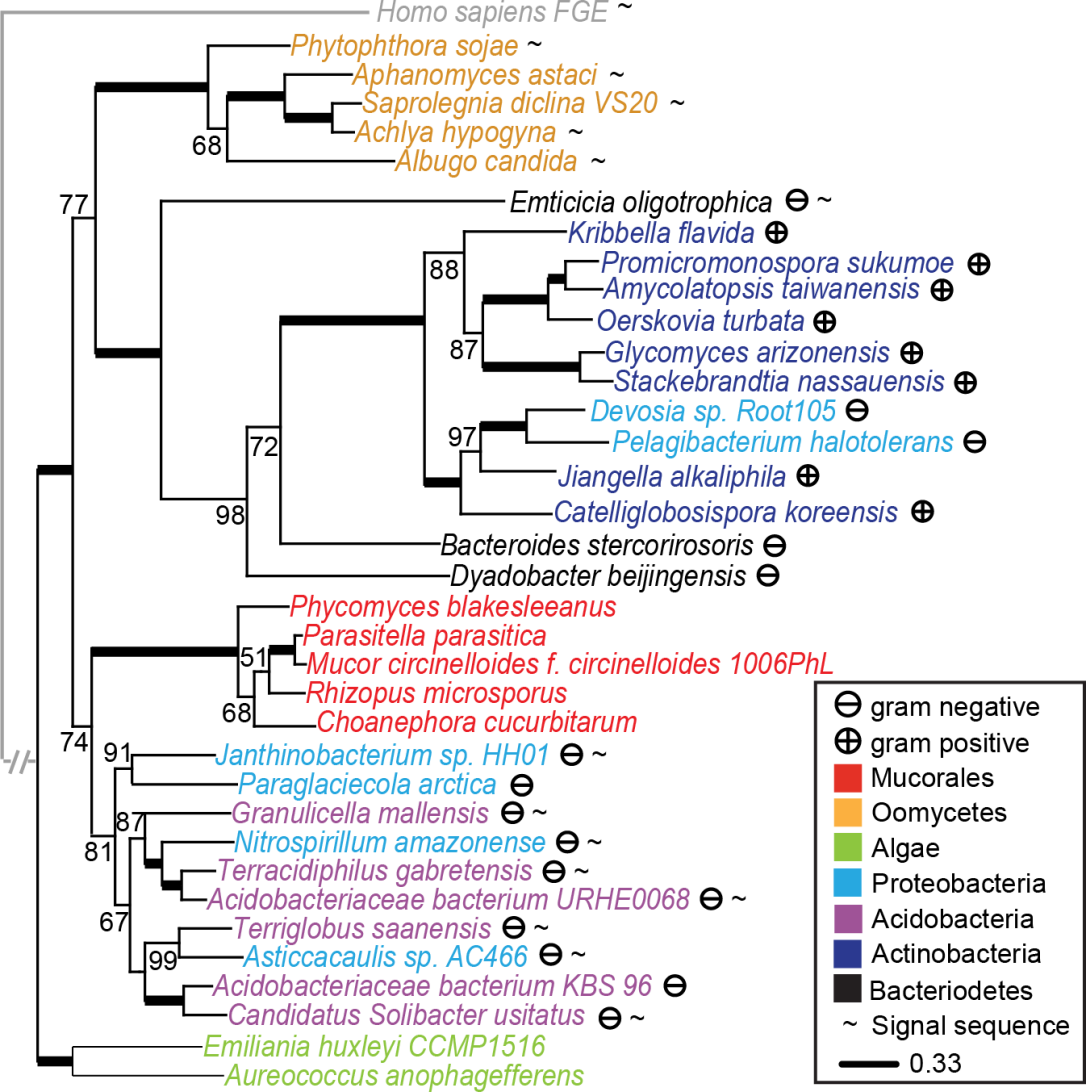
The OCTIN phylogeny indicates multiple HGT events. The OCTIN ML phylogenetic tree supports bacteria-Mucorales, bacteria-Oomycetes, and bacteria-bacteria HGTs. Support values greater than 50 are shown as node labels. Values of 100 are represented by thick horizontal lines. The tree is rooted with a distant homolog, human FGE. Sequences with predicted N-terminal signal sequences, and gram-negative and gram-positive bacteria are marked with the indicated symbols. The various taxa are color-coded according to the legend. The full ML tree constructed from 127 OCTIN sequences is shown in S3A Fig. FGE, formylglycine-generating enzyme; HGT, horizontal gene transfer; ML, maximum likelihood; OCTIN, octahedral crystal matrix protein.

The sporadic distribution of OCTIN in eukaryotes (Fig 1E) could be explained by an early origin followed by extensive gene loss. However, both maximum likelihood (ML) and Bayesian analyses provide strong support for independent acquisition of OCTIN by the Mucoromycotina and Oomycetes through HGT from bacteria. In the ML tree, the Mucorales and Oomycetes each have a distinct sister bacterial group (Figs 2 and S3 and S4), while in the Bayesian tree, the Mucorales are nested within a clade of acido- and proteobacteria (S5 Fig). Enforcing eukaryote monophyly on the ML OCTIN phylogeny results in a topology significantly less likely than the unconstrained phylogeny as judged by the Shimodaira’s Approximately Unbiased (AU) test (*p*-value = 0.021, S1 Table). The trees further suggest HGT among bacteria: acidobacteria and proteobacteria, as well as actinobacteria and proteobacteria, are interspersed to form distinct well-supported monophyletic groups (Figs 2 and S3, S4 and S5), while the constrained topology consistent with vertical transmission is significantly less likely (AU test p-value = 0.009, S2 Table). OCTIN is found in a large number of species in deep branching clades in the proteobacteria and acidobacteria (S6 and S7 Figs), suggesting an ancient origin in bacteria. Together, the phylogenetic analyses support an origin for the gravity-sensing protein crystal through HGT from a gram-negative bacterium.

**Fig 3:**
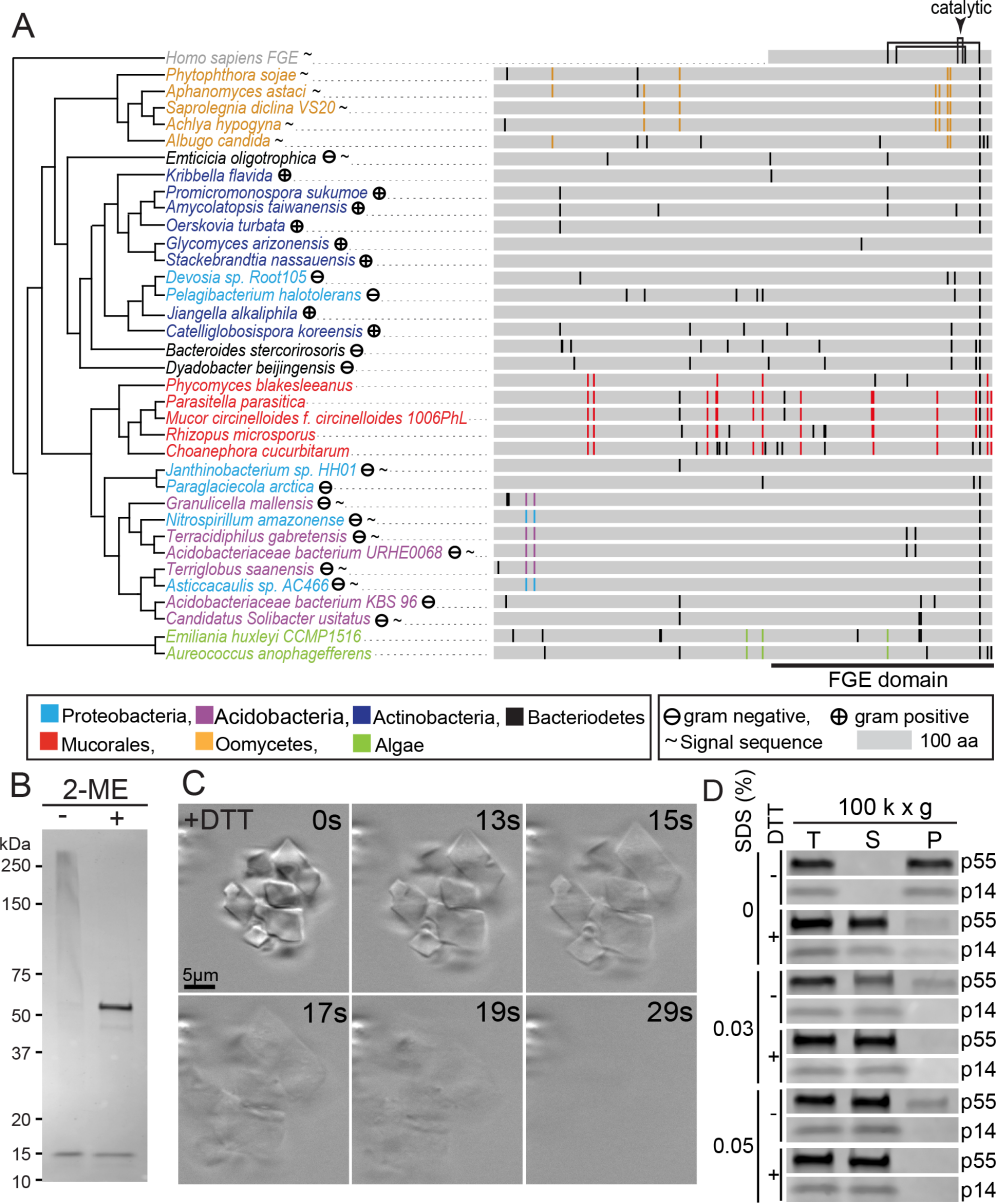
The *Phycomyces* OCTIN crystal lattice is stabilized by intermolecular disulphide bonds. (A) Conservation of cysteine residue position shown by sequence alignment. Positions exhibiting clade-specific conservation are shown in the color of the species to which they correspond. The positions of other cysteine residues are shown in black. Connected lines above the human FGE represent cysteine pairs that form disulphide bonds. The arrowhead indicates the catalytic cysteine pair. The FGE domain is indicated by the horizontal black bar. (B) Crystal-enriched fraction separated by SDS-PAGE in the presence (+) and absence (−) of the reducing agent 2-ME. P55, but not p14, migrates as a high-molecular–weight smear in the absence of 2-ME. Note that p46 also shifts in the absence of 2-ME, suggesting that it is a processing variant of p55. (C) Stills taken from a video recording the disassembly of *Phycomyces* OCTIN crystals by the reducing agent DTT (S1 Movie). (D) Synergistic disassembly of *Phycomyces* OCTIN crystals by SDS and DTT. While SDS alone is sufficient to completely shift p14 to the supernatant after centrifugation at 100,000 x g, only the combination of SDS and DTT has the same effect on p55. 2-ME, 2-Mercaptoethanol; DTT, dithiothreitol; FGE, formylglycine-generating enzyme; OCTIN, octahedral crystal matrix protein; P, pellet; S, supernatant; SDS, sodium dodecyl sulfate; T, total.

The OCTIN C-terminus contains a full-length formylglycine-generating enzyme (FGE) domain (Fig 3A). In metazoans, FGE catalyzes the oxidation of cysteine to Cα-formylglycine to activate sulfatase enzymes in the endoplasmic reticulum (ER). In humans, its loss-of-function causes the fatal genetic disorder multiple sulfatase deficiency (MSD) [30]. Alignment between OCTIN from diverse species and human FGE reveals high overall sequence conservation, with many residues mutated in MSD being conserved in the OCTIN FGE domain. However, key FGE catalytic cysteines are absent in OCTIN sequences, suggesting that OCTIN does not function in sulfatase activation (S8 Fig). Interestingly, many other bacterial FGE domain-containing proteins lack FGE catalytic residues, and like OCTIN, have N-terminal sequence extensions (S9 Fig). In some cases, these extensions show similarity to known domains, which include Kinase, Caspase, DinB, NATCH, and PEGA domains. DinB-FGE has been shown to function as a sulfoxide synthase. This activity depends on DinB catalytic residues that form contacts with the FGE domain [31,32]. Together, these data identify a bacterial superfamily of OCTIN-related proteins. The extent to which these function through structural or enzymatic mechanisms remains to be determined.

The position and number of OCTIN cysteine residues show significant variation between the diverse OCTIN-containing clades. However, within clades, cysteine residues can be well conserved (Fig 3A), suggesting that they tailor OCTIN to its taxa-specific functions. When *Phycomyces* crystals are analyzed by SDS-PAGE under non-reducing conditions, p55 shifts to a high-molecular–weight species that migrates as smear around 250 kDa. By contrast, the migration of p14 is unchanged. These data indicate that p55 forms a disulphide-bonded sub-assembly (Fig 3B). Rapid swelling and disintegration of crystals upon treatment with DTT (dithiothreitol) reveal the importance of disulphide bonds for crystal lattice stability. (Fig 3C and S1 Movie). Centrifugation confirms this effect—p55 and p14 are pelleted by centrifugation at 100,000 x *g*, whereas DTT treatment shifts both into the supernatant fraction. Together, these data further show that p14 associates with p55 through non-covalent interactions. Crystals also swell and dissolve upon addition of the protein denaturant sodium dodecyle sulfate (SDS) (S2 Movie). Neither DTT nor SDS fully solubilizes p55. However, when combined, they synergize to promote disassembly (Fig 3D). Together, these data show that disulphide-bonded p55 sub-assemblies form a crystal lattice through additional non-covalent interactions. p14 is physically associated with the p55 lattice. However, its role in stabilizing this structure is unclear.

**Fig 4:**
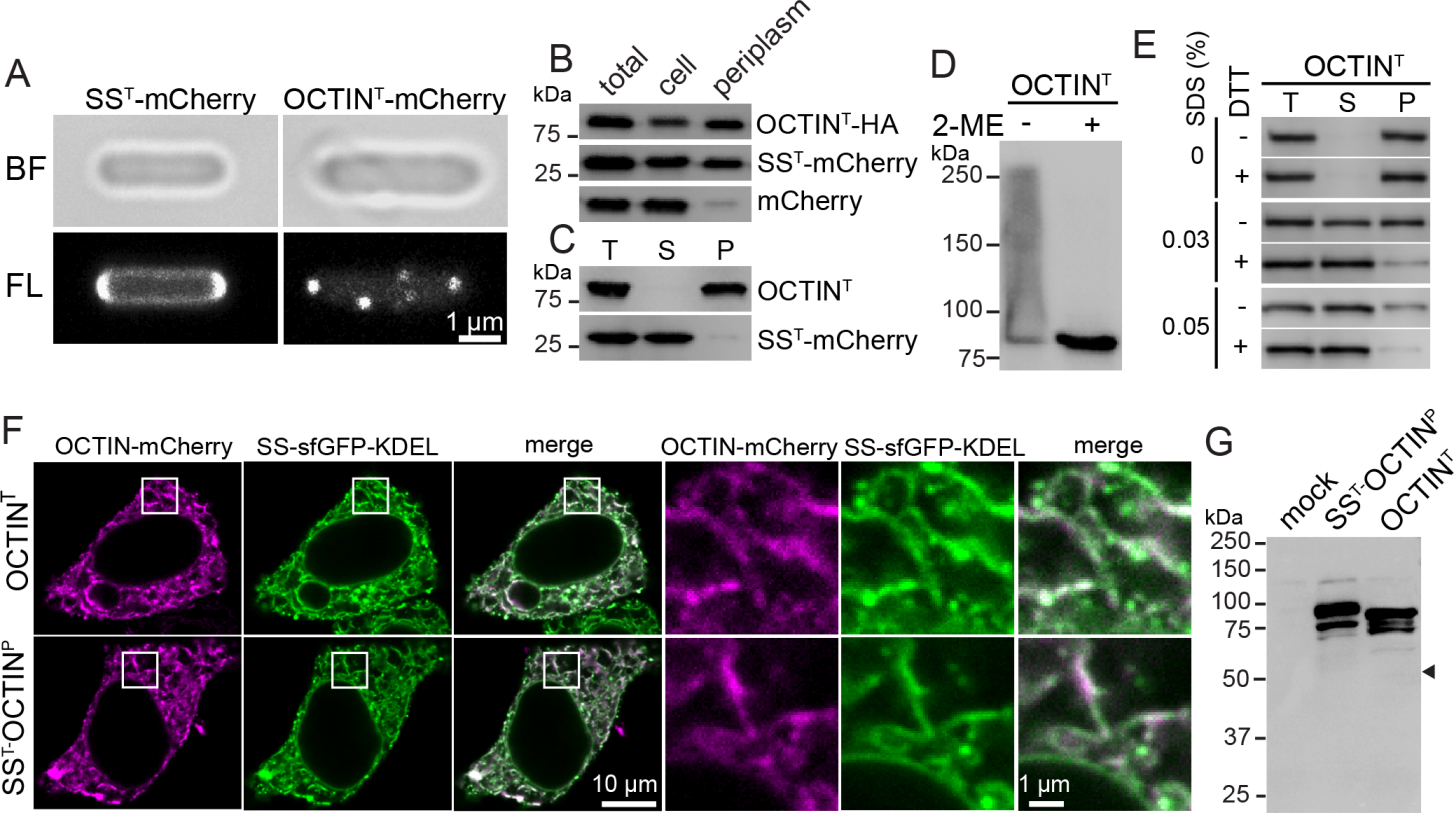
Localization and assembly of bacterial and *Phycomyces* OCTIN upon ectopic expression. (A) *Terriglobus* OCTIN assembles into patches in the periplasm. The *Terriglobus* OCTIN signal sequence fused to the N′-terminus of mCherry (SS^T^-mCherry) is localized in a ring around the cell periphery, while a full-length OCTIN^T^-mCherry fusion protein is localized in patches. (B) Western blotting shows the enrichment of SS^T^-mCherry and OCTIN^T^-HA in an isolated periplasmic fraction. Cytoplasmic mCherry serves as a control for contamination of the periplasmic fraction through cell lysis. (C) OCTIN^T^-HA assembles into high-order oligomers. Periplasmic OCTIN^T^-HA, but not SS^T^-mCherry is pelleted by centrifugation at 100,000 x g. (D) OCTIN^T^ forms intermolecular disulphide bonds. OCTIN^T^-HA migrates as a high-molecular–weight smear in the absence (−) but not presence (+) of 2-ME. (E) As with *Phycomyces* OCTIN crystals (Fig 3D), SDS and DTT synergize to promote the disassembly of OCTIN^T^ oligomers. (F) OCTIN^T^-mCherry and SS^T^-OCTIN^P^-mCherry are targeted to the ER upon expression in mammalian cells. The ER is defined by sfGFP with an N-terminal SS and C′-terminal ER retention signal (KDEL). (G) Western blotting for OCTIN^T^-HA and SS^T^-OCTIN^P^-HA expressed in mammalian cells shows that SS^T^-OCTIN^P^ does not undergo proteolytic processing in the ER. Arrowhead indicates the expected position of p55. 2-ME, 2-Mercaptoethanole; BF, brightfield; DTT, dithiothreitol; ER, endoplasmic reticulum; FL; Fluorescence; OCTIN, octahedral crystal matrix protein; P, pellet; S, supernatant; SDS, sodium dodecyl sulfate; sfGFP, superfolder GFP, SS, signal sequence; T, total.

The origin of a gravity-sensing crystal through HGT from a gram-negative bacterium raises the important question of how bacterial OCTIN might be predisposed to this function. Bacteria descended from the likely *octin* donor are not currently genetically manipulable. To investigate this question, we expressed OCTIN from the gram-negative acidobacterium *Terriglobus saanensis* (OCTIN^T^) in *Escherichia coli*. OCTIN^T^ encodes a predicted signal sequence (SS^T^) and a SS^T^-mCherry fusion protein is targeted to the periplasm as indicated by a fluorescent ring around the cell periphery. By contrast, a full-length OCTIN^T^-mCherry fusion protein produces punctate fluorescence at the cell periphery (Fig 4A). Both proteins are released upon lysis of the outer membrane, indicating that they are indeed periplasmic (Fig 4B). However, only OCTIN^T^ can be pelleted by centrifugation, suggesting that patches seen by fluorescence represent stable high-order oligomers (Fig 4C). Non-reducing SDS-PAGE shows that like *Phycomyces* OCTIN, OCTIN^T^ forms intermolecular disulphide bonds (Fig 4D). Furthermore, as with *Phycomyces* OCTIN, SDS and DTT synergize to promote OCTIN^T^ oligomer disassembly (Fig 4E). Compared with *Phycomyces* OCTIN, DTT alone has little effect, suggesting that these assemblies rely more on non-covalent interactions. Nevertheless, these data support a related underlying mechanism of self-assembly for *Phycomyces* and bacterial OCTIN.

*Phycomyces* sporangiophores are approximately 100 μm in diameter [33] and OCTIN crystals have an average edge length of 5 μm [27]. By contrast, *octin*-containing bacteria whose sizes are known have diameters ranging from 0.3 to 0.8 μm [34–39]. To the best of our knowledge, bacterial gravitropism has not been observed. Moreover, assuming an OCTIN assembly size of 1 μm or less, and taking into account cytoplasmic viscosity [40], the density of OCTIN crystals [41], and the bacterial cytoplasm [41], an estimation of sedimentation velocity based on Stokes’ law indicates that bacterial OCTIN oligomers would be too small to function as gravity sensors. The low ratio of particle movement by gravitational force relative to Brownian motion (Péclet number, [42]) for oligomers in this size range further demonstrates that their movements would be dominated by thermal fluctuations (S10 Fig and S1 Text) [22]. While the function of OCTIN in bacteria remains unknown, its ability to form high-order oligomers is likely to have predisposed neo-functionalization towards a role in gravity sensing in the Mucorales. This is likely to have required the accumulation of mutations relating to crystal lattice assembly, vacuole targeting, and proteolytic processing. If primitive assemblies were too small to function as gravity sensors (S10 Fig), what factors could account for the retention of *octin*? *Phycomyces* OCTIN crystals are found in clusters (Fig 1A), which increases their effective size and sedimentation velocity [24]. Similarly, early OCTIN oligomers could have acted as sensors by clustering. Other scenarios involving neutral selection or another function could also have played a role in the evolutionary transition. In the latter scenario, we note that presently available information does not preclude an enzymatic activity for OCTIN.

The periplasm of gram-negative bacteria and the eukaryotic secretory pathway are both oxidizing environments that share a related machinery for translocation of proteins from the cytoplasm [43]. Indeed, OCTIN^T^-mCherry is targeted to the ER when expressed in mammalian tissue culture cells (Fig 4F). To determine whether *Phycomyces* OCTIN (OCTIN^P^) can self-assemble upon heterologous expression, we expressed an ER-targeted version in mammalian cells. This version of OCTIN co-localizes with an ER lumenal marker, but does not display a punctate signal, suggesting an absence of self-assembly. Western blotting further shows an absence of proteolytic processing (Fig 4G). This indicates that OCTIN crystal assembly is likely to require taxa-specific processing activities. Many vacuolar hydrolases are synthesized as auto-inhibited precursors, which are activated upon delivery to the vacuole through processing by resident proteases [44]. We speculate that the region between p14 and p55 functions to inhibit crystal lattice formation through an analogous mechanism (see S11 Fig for a model of OCTIN assembly). *Phycomyces* has yet to be transformed [33], and this limits its use as a model system. Thus, understanding the control of crystal assembly will require the identification of OCTIN processing factors and reconstitution in a genetically amenable model system.

Phylogenetic analyses strongly support the acquisition of bacterial OCTIN by the Mucorales ancestor through HGT (Figs 2 and S3, S4 and S5). Through its signal sequence, this protein would have been targeted to the endomembrane system (Fig 4F). In this context, the size constraint on OCTIN oligomers was relieved, allowing eventual increase in assembly scale and emergence of the gravity-sensing novelty. The case of OCTIN exemplifies how HGT of a protein undergoing high-order assembly can lead to a novel function that emerges depending on a combination of cellular potentialities and physiological imperatives.

## Methods

### OCTIN identification and phylogenetic analyses

*P*. *blakesleeanus* wild-type strain NRRL155 [25] and crystal-less mutant strain C2 [24] were grown as previously described [41]. Octahedral crystals were purified as previously described [27]. Bands corresponding to p14 and p55 were analyzed by mass spectrometry and Edman degradation (Alphalyse A/S, Odense, Denmark). Peptides p14, p46, and p55 identified the same *P*. *blakesleeanus* protein (National Center for Biotechnology Information [NCBI] accession: XP_018295118.1). The search for OCTIN homologs was performed with BLASTP [45] against the NCBI nonredundant database [46] using the OCTIN-specific N-terminal domain (amino acids 1–500) as the query. HMMER3 [47] was used to confirm the presence of the FGE domain (PF03781) [48] in BLAST hits. The accessions of these hits are reported in S3 Table.

The extended bacterial species trees (S2 and S9 Figs) were constructed based on a previously reported microbial phylogeny [49]. The original tree, which contains multiple strains from the same species, was pruned to retain 1 strain per species whose annotated genome is available in the NCBI Reference Sequence Database (RefSeq, ftp.ncbi.nlm.nih.gov/refseq/). PhyloPhlAn [49] was used to insert additional *octin*-possessing species that are not present in the original tree (S4 Table). All other species trees (S4, S6 and S7 Figs) were constructed from 400 conserved protein sequences by PhyloPhlAn using RefSeq bacterial proteomes. The presence of signal sequence was predicted using Phobius [50]. Phylogenetic trees were visualized with ETE3 [51].

To construct OCTIN protein trees (Figs 2 and S3 and S5), OCTIN sequences from the NCBI reference protein database were used. MAFFT [52] with the option E-INS-i was used to obtain sequence alignments, which were trimmed using Trimal [53] at a gap threshold of 70%. ML bootstrap analysis was performed with RAxML [54] using the automatic bootstrapping option [55] (300 replicates) and the PROTGAMMAILGX model as suggested by ProtTest [56]. The human FGE sequence, which serves as the outgroup (Fig 2), was placed on the ML tree a posteriori using the RAxML option -f v [57]. Bayesian trees were constructed using MrBayes [58], run with 12 chains, temperature 0.05, sampling every 500th generation for 300,000 generations. Convergence was assessed using RWTY [59]. The ML and Bayesian phylogenies, as well as the matrix used to derive them are accessible under the identifier S22330 at TreeBASE (https://treebase.org/). To compare the ML trees with and without the monophyly constraint, the best-scoring tree with monophyly constraint was constructed using RAxML with the same parameters specified above for the construction of unconstrained trees. Phylogenetic hypothesis testing using the resampling estimated log-likelihood (RELL) test, Shimodaira–Hasegawa (SH) test, Kishino–Hasegawa (KH) test, and AU test was then performed with the PAML package ‘codeml’ [60] and CONSEL [61].

### Identification of FGE domain-containing proteins

The search for FGE domain-containing proteins was performed with HMMER3 [47], using the FGE alignment (PF03781) downloaded from http://pfam.xfam.org. The search was performed on RefSeq proteomes of species present in the bacterial phylogeny shown in S2 Fig. Sequences containing at least 100 amino acids upstream of the FGE domains were selected. Annotated domains within these sequences were identified using the hmmscan function of HMMER3 [47]. Homologs of the gliding motility protein GldK, whose N-terminal domain is not annotated, were manually added based on similiarity to the known GldK sequence from *Flavobacterium johnsoniae* (NCBI accession: AAW78679.1).

### Recombinant OCTIN expression

*T*. *saanensis octin* was codon-optimized for expression in *E*. *coli* and the synthetic sequence was obtained from Genscript. Full-length *octin*^*P*^ was amplified by reverse transcription polymerase chain reaction (RT-PCR) from *Phycomyces* sporangiophore total RNA. *Octin* sequences and mCherry fusions were integrated into the pETDuet-1 vector (Novagen, cat #71146) for transformation in *E*. *coli* strain HMS174 (Novagen, cat #69453). Primers used in generating the expression plasmids are listed in S5 Table. *E*. *coli* periplasmic extract was obtained following a previously described protocol [62] with modifications. The induced culture was centrifuged at 2,500 x *g* and 4 ^o^C for 10 minutes. The pellet was then gently resuspended in ice-cold PE buffer (20% sucrose, 1 mM EDTA, 50 mM Tris pH 7.4) and placed on a nutating mixer at 4 ^o^C for 15 minutes. This was followed by centrifugation at 2,500 x *g* and 4°C for 10 minutes. The supernatant was transferred to a clean tube and supplemented with Halt protease and phosphatase inhibitor cocktail (ThermoFisher 78440). This extract was aliquoted and flash-frozen for disassembly assays and western blot. OCTIN^T^ and mCherry variants were detected by western blotting using horseradish-peroxidase–conjugated rat anti-HA antibodies (ROCHE, cat# 12013819001) or mouse anti-mCherry (SAB2702286 SIGMA) and secondary goat anti-mouse IgG (SAB4600004 SIGMA). Blot images were acquired using the ChemiDoc Touch Imaging System (Bio-Rad).

### OCTIN crystal and bacterial oligomer disassembly

Crystals suspended in Tris-buffered saline buffer (TBS; 10 mM Tris pH 7.2, 150 mM NaCl) were mounted on a microscope slide. DTT or SDS was added to one side of the coverslip to a final concentration of 50 mM or 0.1%, respectively. Crystal disassembly was recorded using an epifluorescence microscope (BX51; Olympus) and a digital camera (Coolsnap HQ; Photometrics) controlled by Metamorph.

Synergistic disassembly of *Phycomyces* crystals (Fig 3D) and bacterial OCTIN oligomers (Fig 4E) by SDS and DTT was performed by incubating the crystals or periplasmic extract with the indicated combinations of SDS and DTT for 30 minutes at 25 ^o^C. This was followed by centrifugation at 100,000 x *g* for 30 minutes at 25 ^o^C. The total sample and the resulting supernatant and pellet fractions were analyzed by SDS-PAGE.

### *E*. *coli* imaging

Overnight cultures of transformed HMS174 cells were diluted into fresh media and allowed to grow to OD600 of 0.7 before induction with 1 mM IPTG. After 4 hours, 5 μl of the suspension was diluted into 1 ml of fresh LB media and 300 μl was placed on a 35-mm microscopy dish (Matek P35G-1.5-10-C) that had been pre-treated with 50 μg/ml poly-D-lysine (Sigma P7886). After 1 hour the media was removed and replaced with 2 ml of fresh media. Imaging was carried out with a Leica SP8 inverted laser-scanning confocal microscope fitted with a white-light laser and 100x lens of numerical aperture (NA) 1.4. Each image is composed of 4 averaged frames taken at 1% laser power at 587-nm excitation with a scan speed of 400 MHz.

### Mammalian cell culture

HeLa cells cultured in 6-well dishes or 8-well chamber slides were transiently transfected with the indicated plasmids using lipofectamine 3000 (ThermoFisher) and cultured for 48 hours before fixing for microscopy or harvesting for western blot analysis. Cells were fixed with 4% Paraformaldehyde (EMS #15700) in phosphate buffered saline (PBS) and then kept in 90% glycerol PBS for imaging. Imaging was carried out using a Leica SP8 fitted with a 63x objective NA of 1.4. The white-light laser was set to 488 nm and 587 nm for GFP and mCherry, respectively. Images are a single *z* plane taken with 8 line averages at 5% laser power, with a scan speed of 200 MHz, 50% gain and a pixel size of 70 nm. To extract protein for western blotting, HeLa cells were lysed in RIPA buffer (50 mM Tris-HCl pH7.4, 150 mM NaCl, 1% Triton-X100, 0.1% Sodium Deoxycholate, 1% SDS) supplemented with Halt protease and phosphatase inhibitor cocktail. Insoluble material was pelleted at 10,000 x *g* and the supernatant fraction boiled in SDS-PAGE loading dye. 10 μg of total cell extract was run per lane. Western blotting was carried out as stated above.

## Supporting information

S1 Fig Cell-type–specific *octin* expression and sequencing of the crystal-less mutant. (A) The full-length *octin* transcript is expressed exclusively in sporangiophores. RNA was extracted from the indicated cell types and subjected to RT-PCR to amplify the indicated cDNAs. The *octin* primers are designed to amplify the entire predicted open reading frame. (B) Premature stop codon in the octin open reading frame of the crystal-less mutant. Chromatograms of WT and crystal-less strains and alignment to the reference sequence (XM_018441888.1). Translated sequence is shown below the corresponding nucleotides. The base substitution resulting in the premature stop codon is highlighted in gray. Numbers on the left represent the starting positions of the nucleotide and protein sequences. This figure was generated with Benchling (benchling.com). RT-PCR, reverse transcription polymerase chain reaction; WT, wild type.
(TIF)

S2 Fig Extended bacterial species tree showing the sporadic distribution of OCTIN-containing species. *Octin*-possessing species are indicated by red bars. OCTIN, octahedral crystal matrix protein.
(TIF)

S3 Fig ML phylogenetic analysis supports OCTIN HGT between bacteria. Bootstrap supports greater than 50 are shown as node labels. The tree is rooted with the human FGE sequence. Values of 100 are represented by thick horizontal lines. Taxa are color-coded according to the legend. FGE, formylglycine-generating enzyme; HGT, horizontal gene transfer; ML, maximum likelihood; OCTIN, octahedral crystal matrix protein.
(TIF)

S4 Fig Bacterial species tree constructed from 400 conserved protein sequences. Shimodaira–Hasegawa support value is shown at the corresponding branch. Taxa are color-coded according to the legend.
(TIF)

S5 Fig The Bayesian inference of the OCTIN phylogeny. (A) Bayesian tree constructed from the same eukaryotic and bacterial OCTIN sequences as shown in S3A Fig. Taxa are color-coded according to the legend. (B) Convergence assessment of the Bayesian OCTIN trees performed using RWTY [59]. OCTIN, octahedral crystal matrix protein.
(TIF)

S6 Fig Distribution of acido- and proteobacterial OCTINs suggests an ancient bacterial origin. High density of OCTIN-containing species in an acidobacterial clade. Species containing OCTIN are shown in purple. The species tree was constructed from 400 conserved protein sequences with annotated genomes. The tree is rooted using proteobacteria whose names are in gray. Shimodaira–Hasegawa branch support values are shown as node labels. OCTIN, octahedral crystal matrix protein.
(TIF)

S7 Fig High density of OCTIN-containing species in the proteobacterial clade Xanthomonadales. Species containing OCTIN are in blue. The Xanthomonadales species tree was constructed from 400 conserved protein sequences with annotated genomes. The tree is rooted using proteobacteria whose names are in gray. Shimodaira–Hasegawa branch support values are shown as node labels. OCTIN, octahedral crystal matrix protein.
(TIF)

S8 Fig Alignment of the FGE domain from the indicated species. Catalytic residues required for sulfatase activation by human FGE are highlighted in red and shown in bold font. Mutations resulting in MSD are shown in parentheses above the alignment. Secondary structural features defined by human FGE crystal structure are identified with black bars and labeled. Cysteine residues colored yellow form an intramolecular disulfide bridge in human FGE and pFGE. Residues associated with calcium binding in FGE and pFGE are shown in blue. Species names are colored according to S3 Fig legend. FGE, formylglycine-generating enzyme; MSD, multiple sulfate deficiency; pFGE, FGE paralog.
(TIF)

S9 Fig The OCTIN superfamily: Distribution of FGE domain-containing protein families in bacteria. The presence of different protein subfamilies is indicated by colored bars. Members of the FGE subfamily possess catalytic residues and do not contain other domains. FGE domain-containing proteins whose N-terminal extension shows similarity to a known domain are color-coded according to the legend. Those containing novel domains are indicated by black bars. All of these lack FGE catalytic cysteine residues. FGE, formylglycine-generating enzyme; OCTIN, octahedral crystal matrix protein.
(TIF)

S10 Fig Estimated sedimentation properties of hypothetical OCTIN assemblies of varying diameters. (A) Sedimentation velocity estimated based on Stokes’ law, taking into account cytoplasmic density and viscosity (S1 Text). The cross indicates the reported sedimentation velocity of *Phycomyces* crystal clusters [26]. The estimated particle size corresponding to this sedimentation velocity is in agreement with actual cluster size [26]. The inset shows estimated sedimentation velocity of sub-micron particles in μm/minute. Grey region indicates the size range of cytoplasmic particles in OCTIN-possessing bacteria, given the cell diameter range of 0.3–0.8 μm (S1 Text). (B) Péclet number of hypothetical OCTIN assemblies. The cross indicates the Péclet number corresponding to the *Phycomyces* crystal cluster documented in reference [26]. Note that thermal fluctuations dominate the movement of assemblies in the size range of bacterial cytoplasmic bodies. OCTIN, octahedral crystal matrix protein.
(TIF)

S11 Fig Model for formation of the OCTIN crystal lattice. The boxed cartoon depicts different regions of full-length OCTIN. The formation of a 3-dimensional protein lattice requires a minimum of 3 intermolecular contacts. For simplicity, the disulphide crosslinked p55 sub-assembly is depicted as a trimer and the lattice is depicted in two dimensions. We speculate that non-covalent contacts required for assembly are shielded by the region between p14 and p55, which is removed by proteolytic processing. The folding and processing events could occur simultaneously or in the opposite order to that depicted. Note that the role of p14 in lattice assembly remains unclear. OCTIN, octahedral crystal matrix protein.
(TIF)

S1 Movie Disintegration of *Phycomyces* OCTIN crystals by DTT. This movie complements Fig 3C. DTT, dithiothreitol; OCTIN, octahedral crystal matrix protein.
(AVI)

S2 Movie Disintegration of *Phycomyces* OCTIN crystals by SDS. This movie complements Fig 3C. OCTIN, octahedral crystal matrix protein; SDS, sodium dodecyl sulfate.
(AVI)

S1 Text Biophysical constraints preclude OCTIN’s function in bacterial gravitropism. OCTIN, octahedral crystal matrix protein.
(PDF)

S1 Table Comparison between the best-scoring ML trees constructed from representative bacterial and eukaryotic OCTINs with and without the eukaryote monophyly constraint. ML, maximum likelihood; OCTIN, octahedral crystal matrix protein.
(DOCX)

S2 Table Comparison between the best-scoring ML tree with and without the acidobacteria and proteobacteria monophyly constraint. ML, maximum likelihood.
(DOCX)

S3 Table NCBI accessions of OCTIN homologs used to construct the OCTIN phylogenies. NCBI, National Center for Biotechnology Information; OCTIN, octahedral crystal matrix protein.
(XLSX)

S4 Table Accession and assembly details of proteomes imputed into the microbial phylogeny. (XLSX)

S5 Table Primers used to generate *octin* constructs. (XLSX)

## Abbreviations

2-ME: 2-Mercaptoethanol
AU: Approximately Unbiased
DTT: dithiothreitol
ER: endoplasmic reticulum
FGE: formylglycine-generating enzyme
HGT: horizontal gene transfer
KH: Kishino–Hasegawa
ML: maximum likelihood
MSD: multiple sulfatase deficiency
NA: numerical aperture
NCBI: National Center for Biotechnology Information
OCTIN: octahedral crystal matrix protein
PBS: phosphate buffered saline
RELL: resampling estimated log-likelihood
RT-PCR: reverse transcription polymerase chain reaction
SDS: sodium dodecyl sulfate
sfGFP: superfolder GFP
SH: Shimodaiara–Hasegawa
SS^T^: predicted *Terriglobus* signal sequence
TBS: Tris-buffered saline buffer
