# Supplementary figures and images for: Evolutionary novelty in gravity sensing through horizontal gene transfer and high-order protein assembly
Source: PLoS Biol. 2018 Apr 24;16(4):e2004920. doi: 10.1371/journal.pbio.2004920 (PMC5915273; doi:10.1371/journal.pbio.2004920)

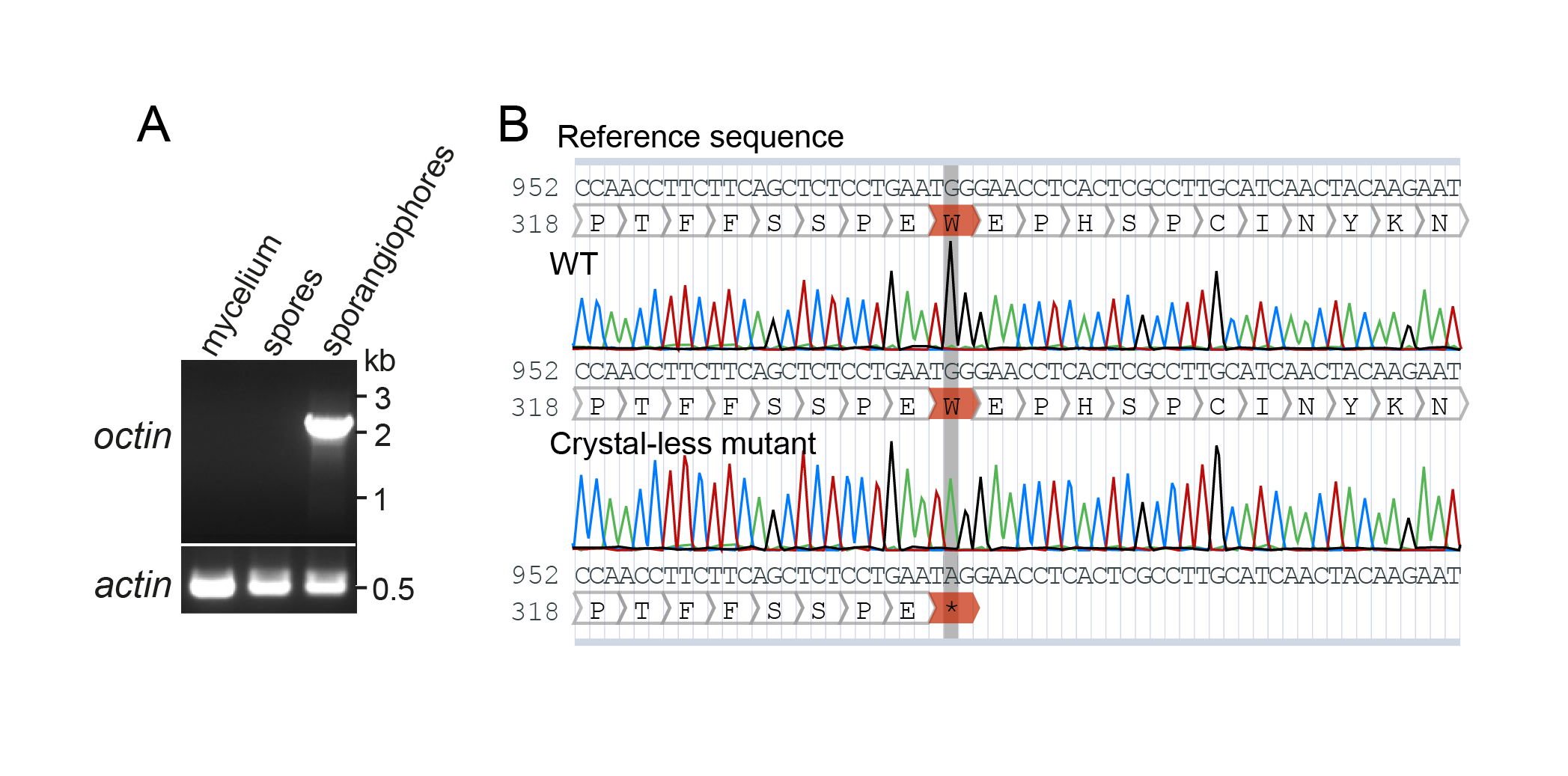

Supplement: S1 Fig — (A) The full-length octin transcript is expressed exclusively in sporangiophores. RNA was extracted from the indicated cell types and subjected to RT-PCR to amplify the indicated cDNAs. The octin primers are designed to amplify the entire predicted open reading frame. (B) Premature stop codon in the octin open reading frame of the crystal-less mutant. Chromatograms of WT and crystal-less strains and alignment to the reference sequence (XM_018441888.1). Translated sequence is shown below the corresponding nucleotides. The base substitution resulting in the premature stop codon is highlighted in gray. Numbers on the left represent the starting positions of the nucleotide and protein sequences. This figure was generated with Benchling (benchling.com). RT-PCR, reverse transcription polymerase chain reaction; WT, wild type. (TIF) [file pbio.2004920.s001.tif]

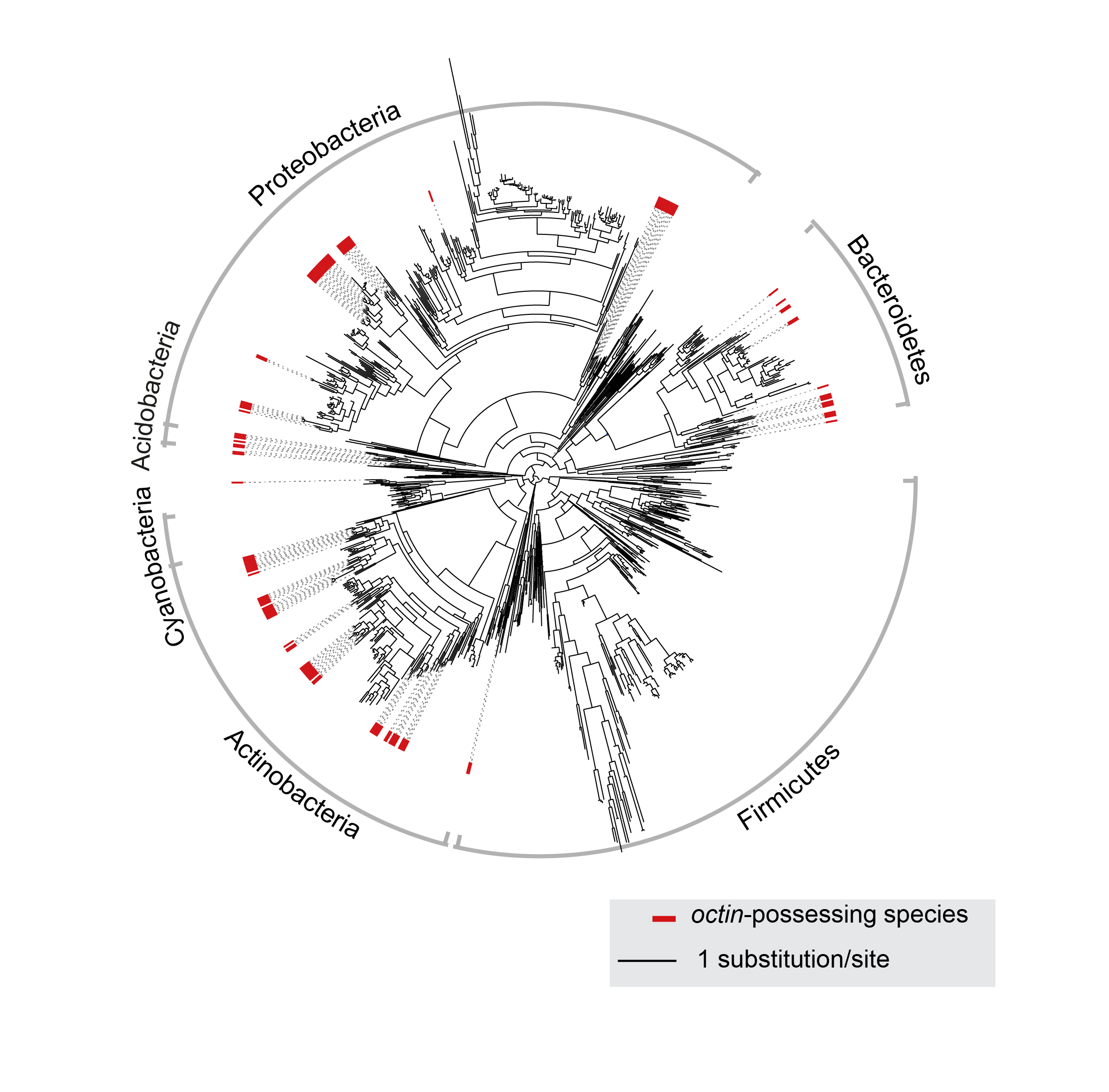

Supplement: S2 Fig — Octin-possessing species are indicated by red bars. OCTIN, octahedral crystal matrix protein. (TIF) [file pbio.2004920.s002.tif]

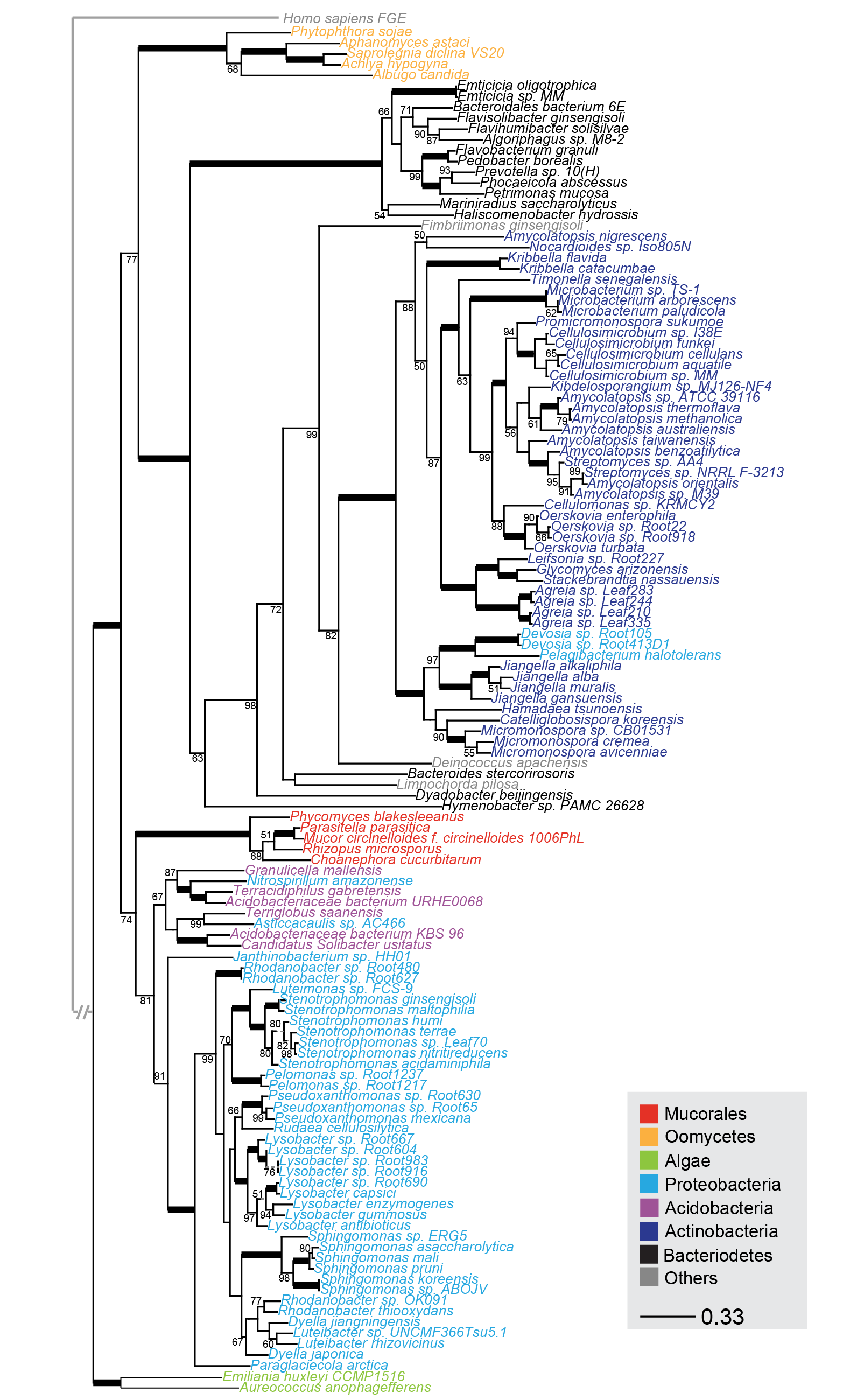

Supplement: S3 Fig — Bootstrap supports greater than 50 are shown as node labels. The tree is rooted with the human FGE sequence. Values of 100 are represented by thick horizontal lines. Taxa are color-coded according to the legend. FGE, formylglycine-generating enzyme; HGT, horizontal gene transfer; ML, maximum likelihood; OCTIN, octahedral crystal matrix protein. (TIF) [file pbio.2004920.s003.tif]

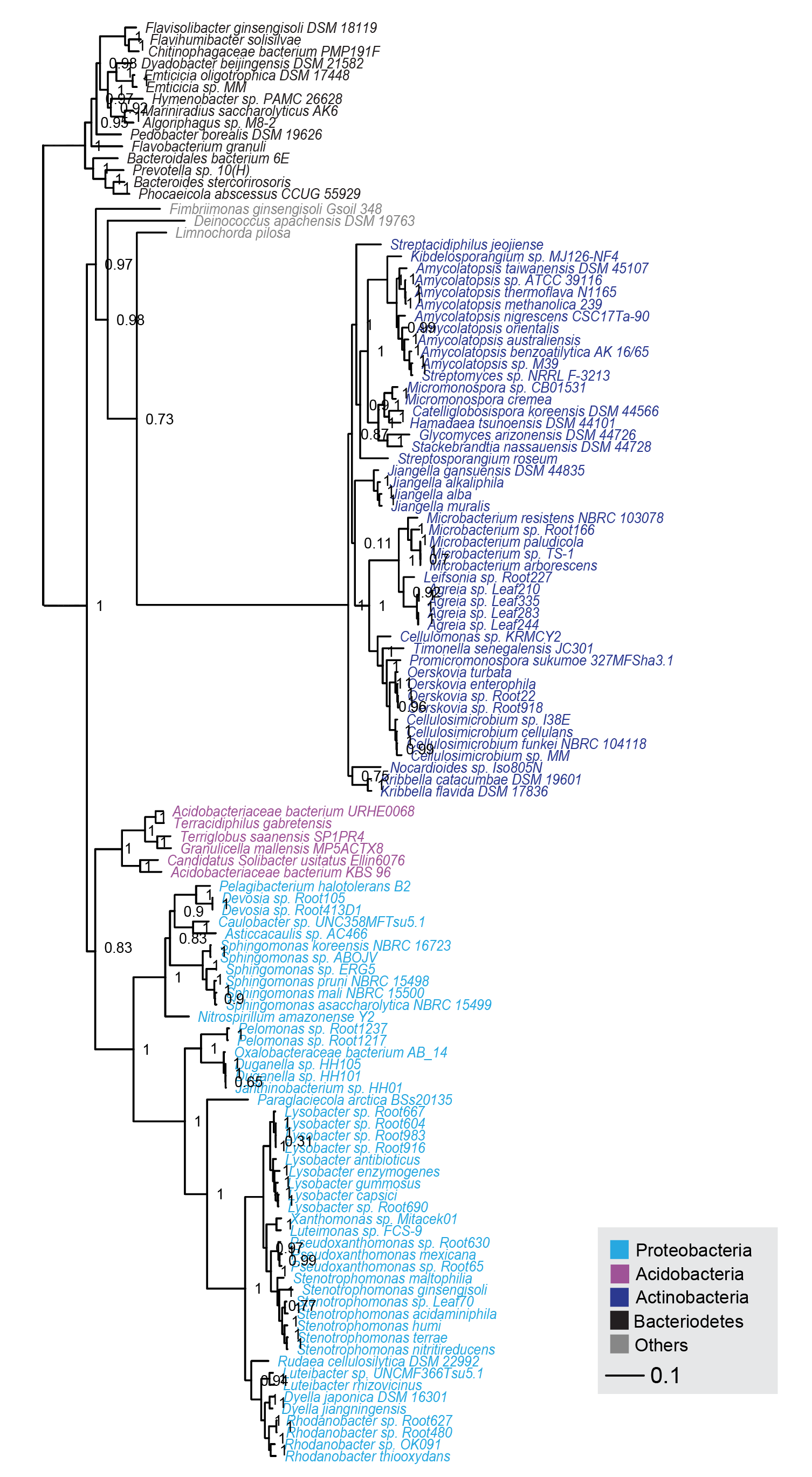

Supplement: S4 Fig — Shimodaira–Hasegawa support value is shown at the corresponding branch. Taxa are color-coded according to the legend. (TIF) [file pbio.2004920.s004.tif]

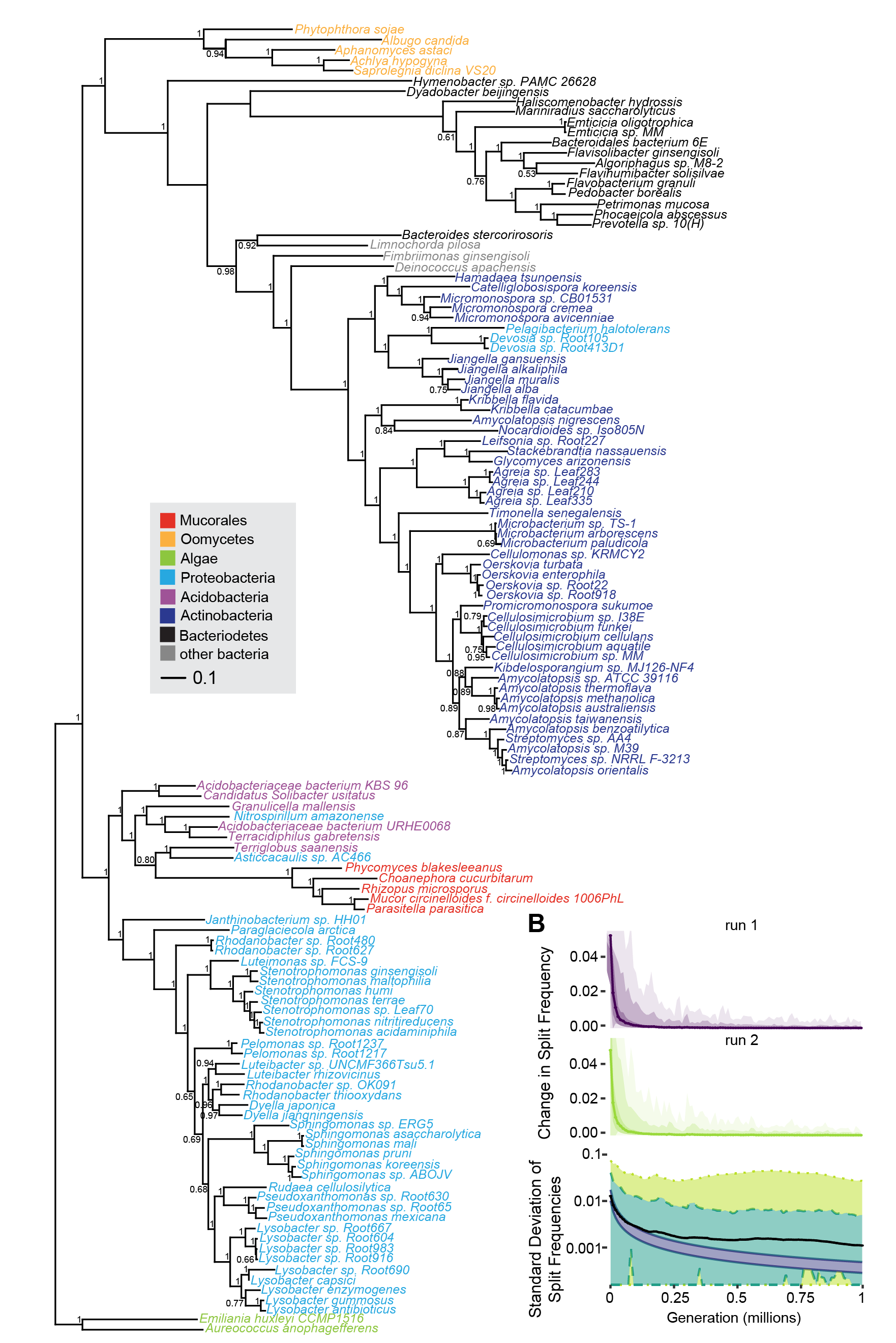

Supplement: S5 Fig — (A) Bayesian tree constructed from the same eukaryotic and bacterial OCTIN sequences as shown in S3A Fig. Taxa are color-coded according to the legend. (B) Convergence assessment of the Bayesian OCTIN trees performed using RWTY [59]. OCTIN, octahedral crystal matrix protein. (TIF) [file pbio.2004920.s005.tif]

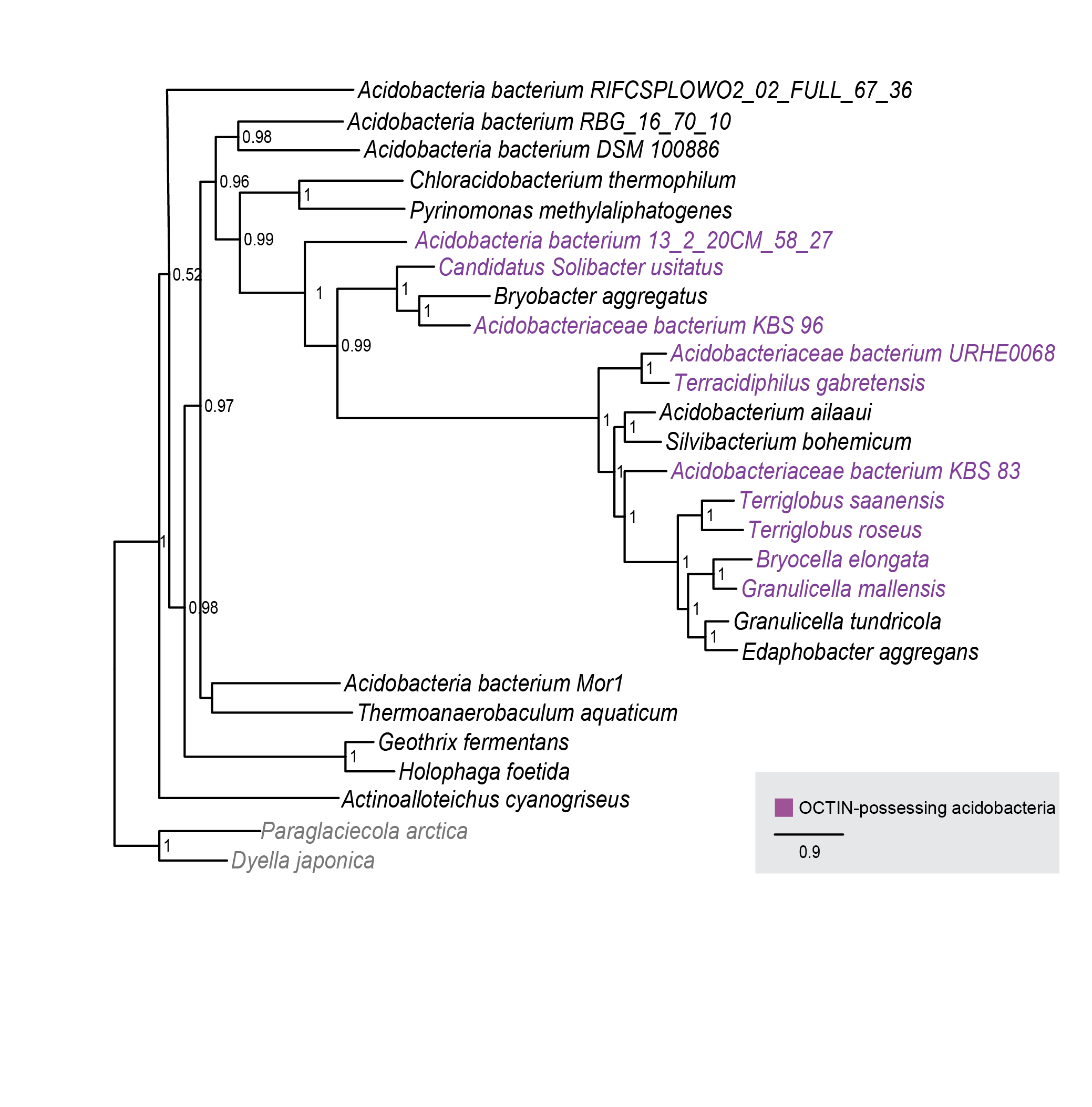

Supplement: S6 Fig — High density of OCTIN-containing species in an acidobacterial clade. Species containing OCTIN are shown in purple. The species tree was constructed from 400 conserved protein sequences with annotated genomes. The tree is rooted using proteobacteria whose names are in gray. Shimodaira–Hasegawa branch support values are shown as node labels. OCTIN, octahedral crystal matrix protein. (TIF) [file pbio.2004920.s006.tif]

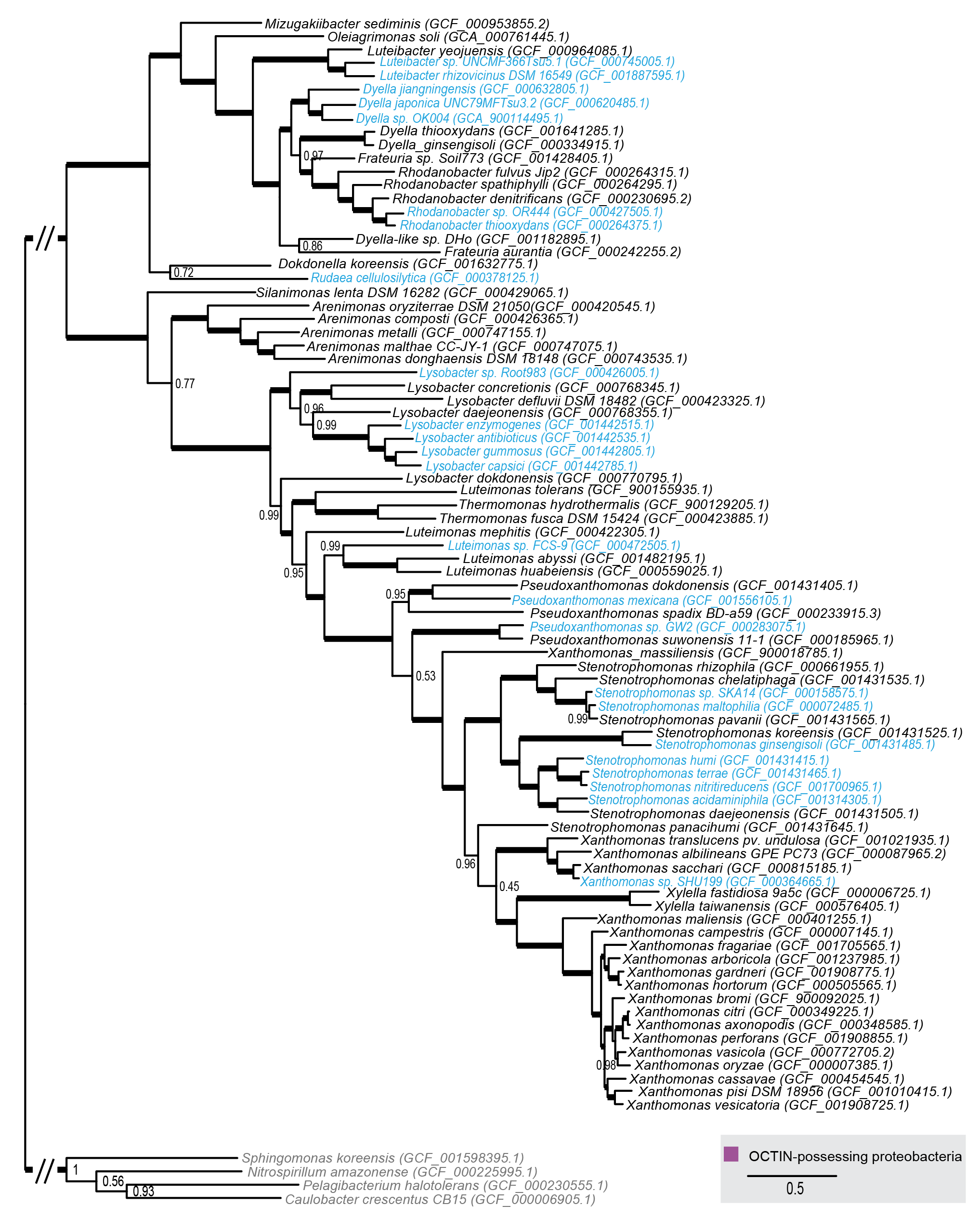

Supplement: S7 Fig — Species containing OCTIN are in blue. The Xanthomonadales species tree was constructed from 400 conserved protein sequences with annotated genomes. The tree is rooted using proteobacteria whose names are in gray. Shimodaira–Hasegawa branch support values are shown as node labels. OCTIN, octahedral crystal matrix protein. (TIF) [file pbio.2004920.s007.tif]

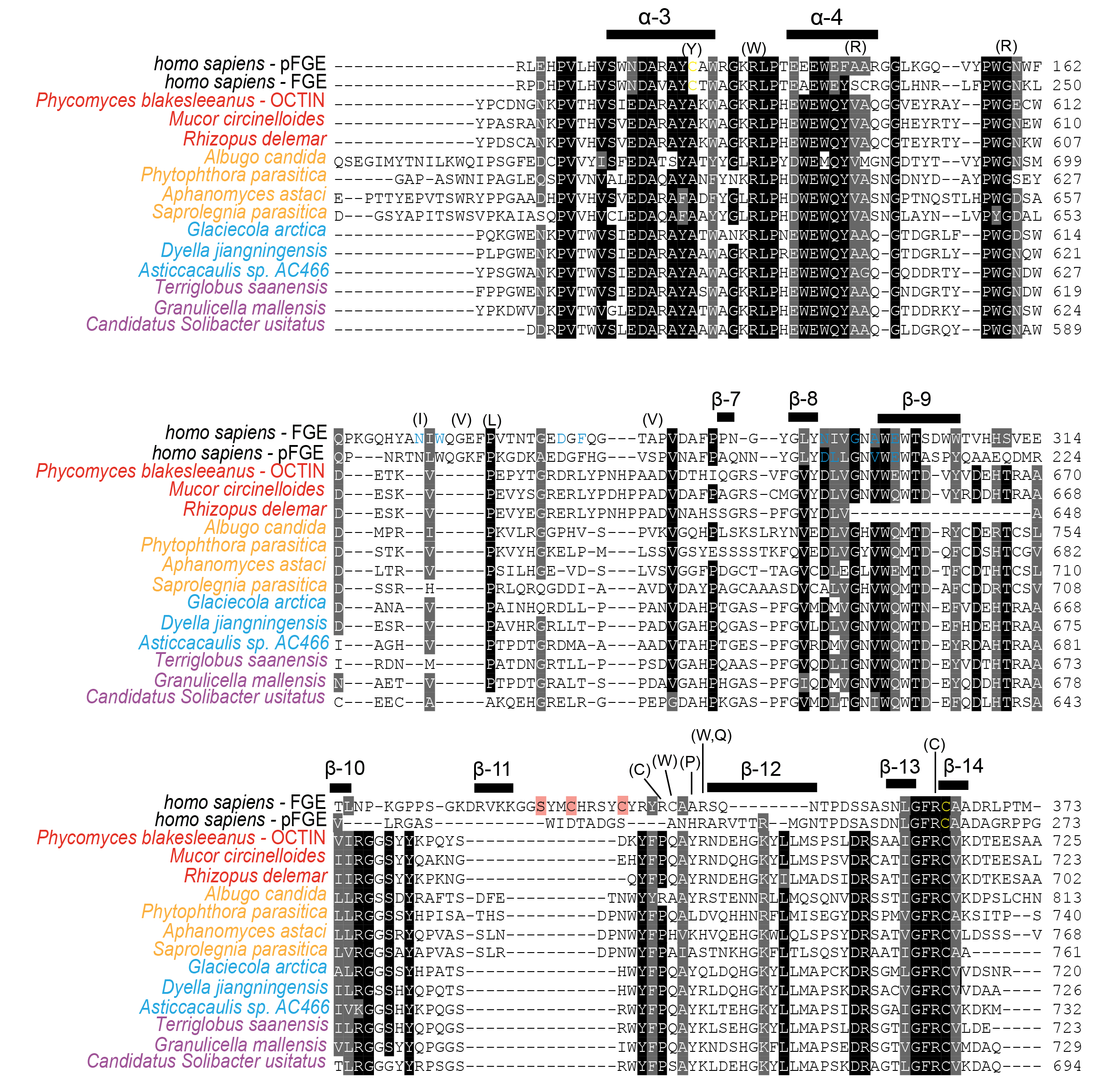

Supplement: S8 Fig — Catalytic residues required for sulfatase activation by human FGE are highlighted in red and shown in bold font. Mutations resulting in MSD are shown in parentheses above the alignment. Secondary structural features defined by human FGE crystal structure are identified with black bars and labeled. Cysteine residues colored yellow form an intramolecular disulfide bridge in human FGE and pFGE. Residues associated with calcium binding in FGE and pFGE are shown in blue. Species names are colored according to S3 Fig legend. FGE, formylglycine-generating enzyme; MSD, multiple sulfate deficiency; pFGE, FGE paralog. (TIF) [file pbio.2004920.s008.tif]

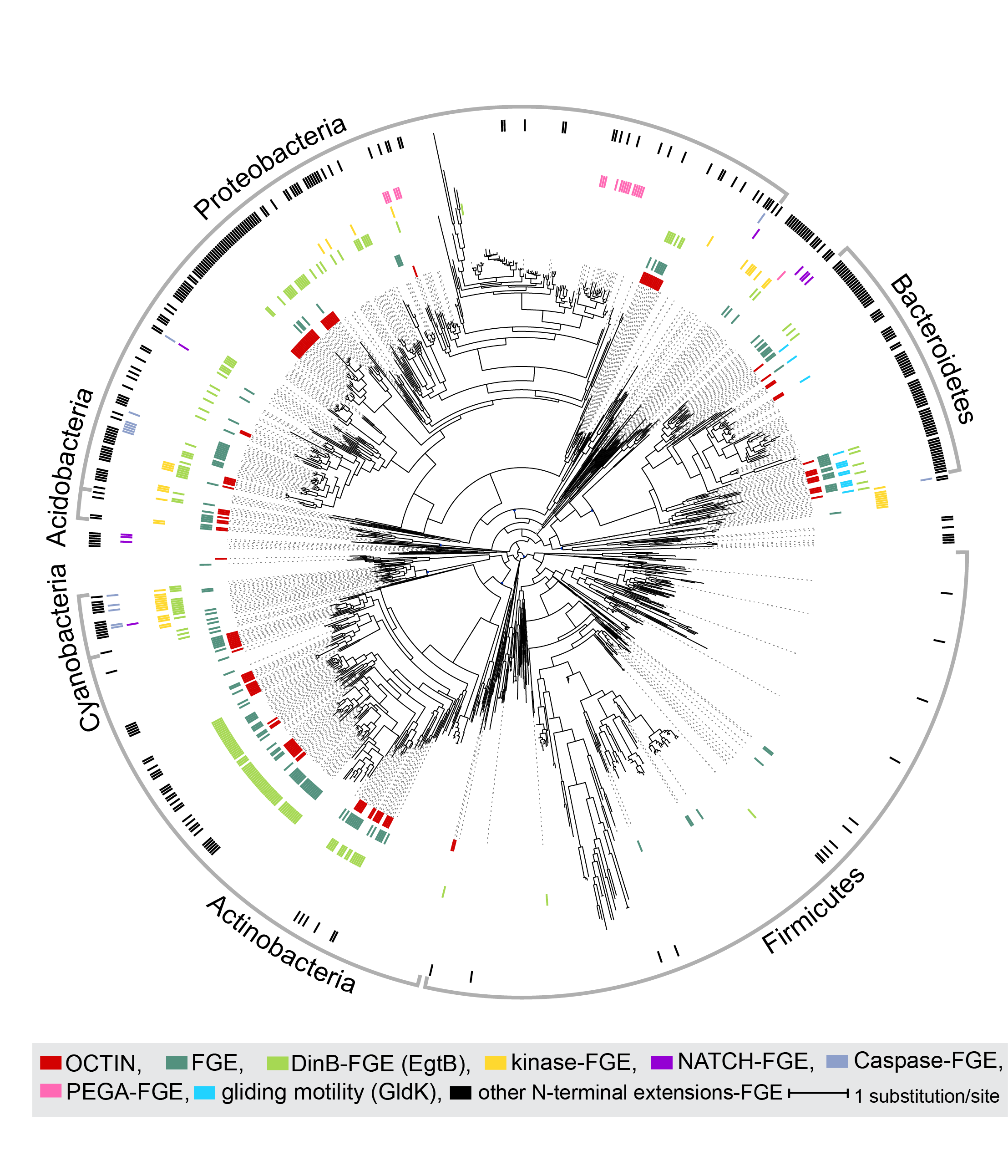

Supplement: S9 Fig — The presence of different protein subfamilies is indicated by colored bars. Members of the FGE subfamily possess catalytic residues and do not contain other domains. FGE domain-containing proteins whose N-terminal extension shows similarity to a known domain are color-coded according to the legend. Those containing novel domains are indicated by black bars. All of these lack FGE catalytic cysteine residues. FGE, formylglycine-generating enzyme; OCTIN, octahedral crystal matrix protein. (TIF) [file pbio.2004920.s009.tif]

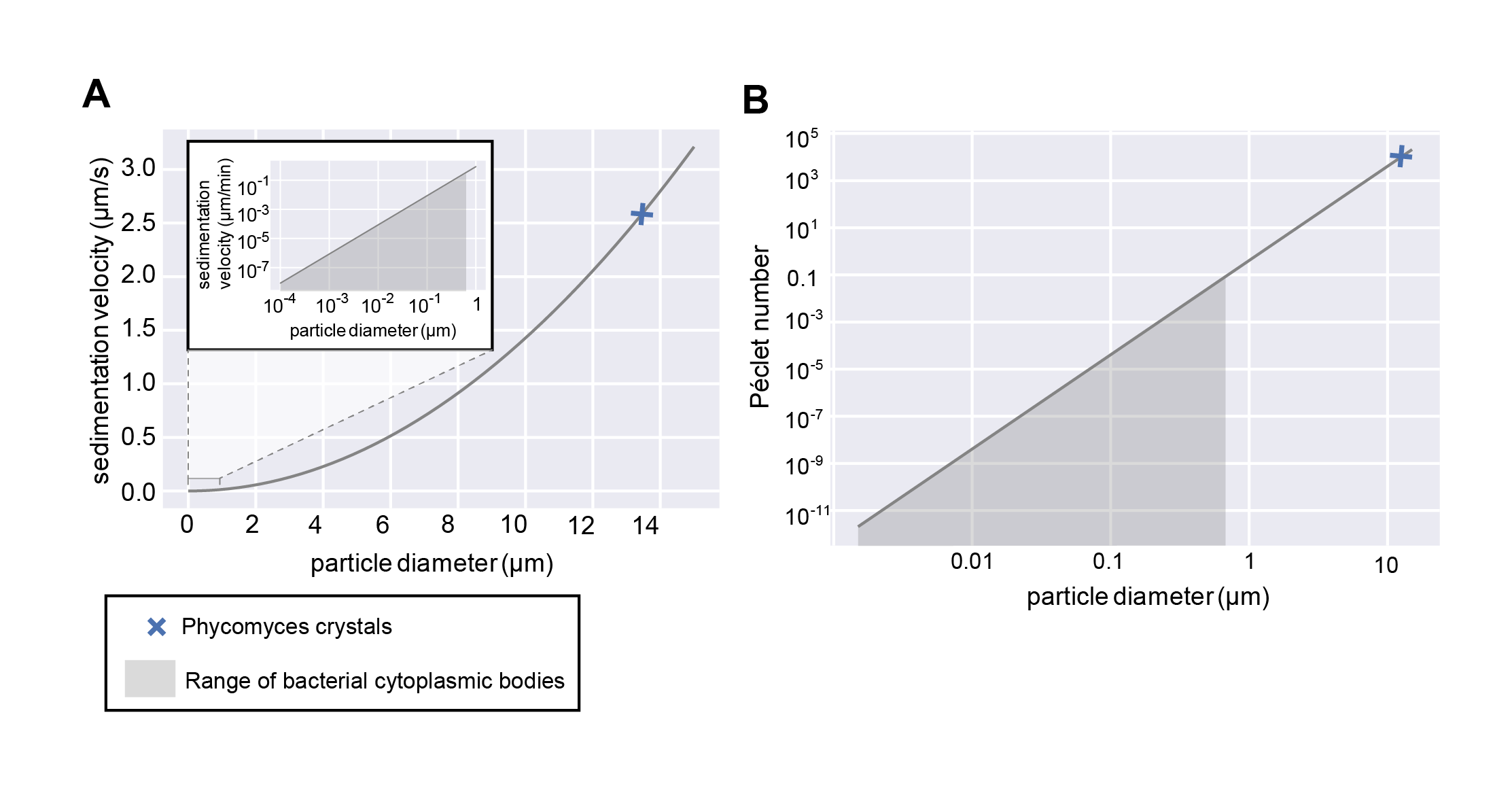

Supplement: S10 Fig — (A) Sedimentation velocity estimated based on Stokes’ law, taking into account cytoplasmic density and viscosity (S1 Text). The cross indicates the reported sedimentation velocity of Phycomyces crystal clusters [26]. The estimated particle size corresponding to this sedimentation velocity is in agreement with actual cluster size [26]. The inset shows estimated sedimentation velocity of sub-micron particles in μm/minute. Grey region indicates the size range of cytoplasmic particles in OCTIN-possessing bacteria, given the cell diameter range of 0.3–0.8 μm (S1 Text). (B) Péclet number of hypothetical OCTIN assemblies. The cross indicates the Péclet number corresponding to the Phycomyces crystal cluster documented in reference [26]. Note that thermal fluctuations dominate the movement of assemblies in the size range of bacterial cytoplasmic bodies. OCTIN, octahedral crystal matrix protein. (TIF) [file pbio.2004920.s010.tif]

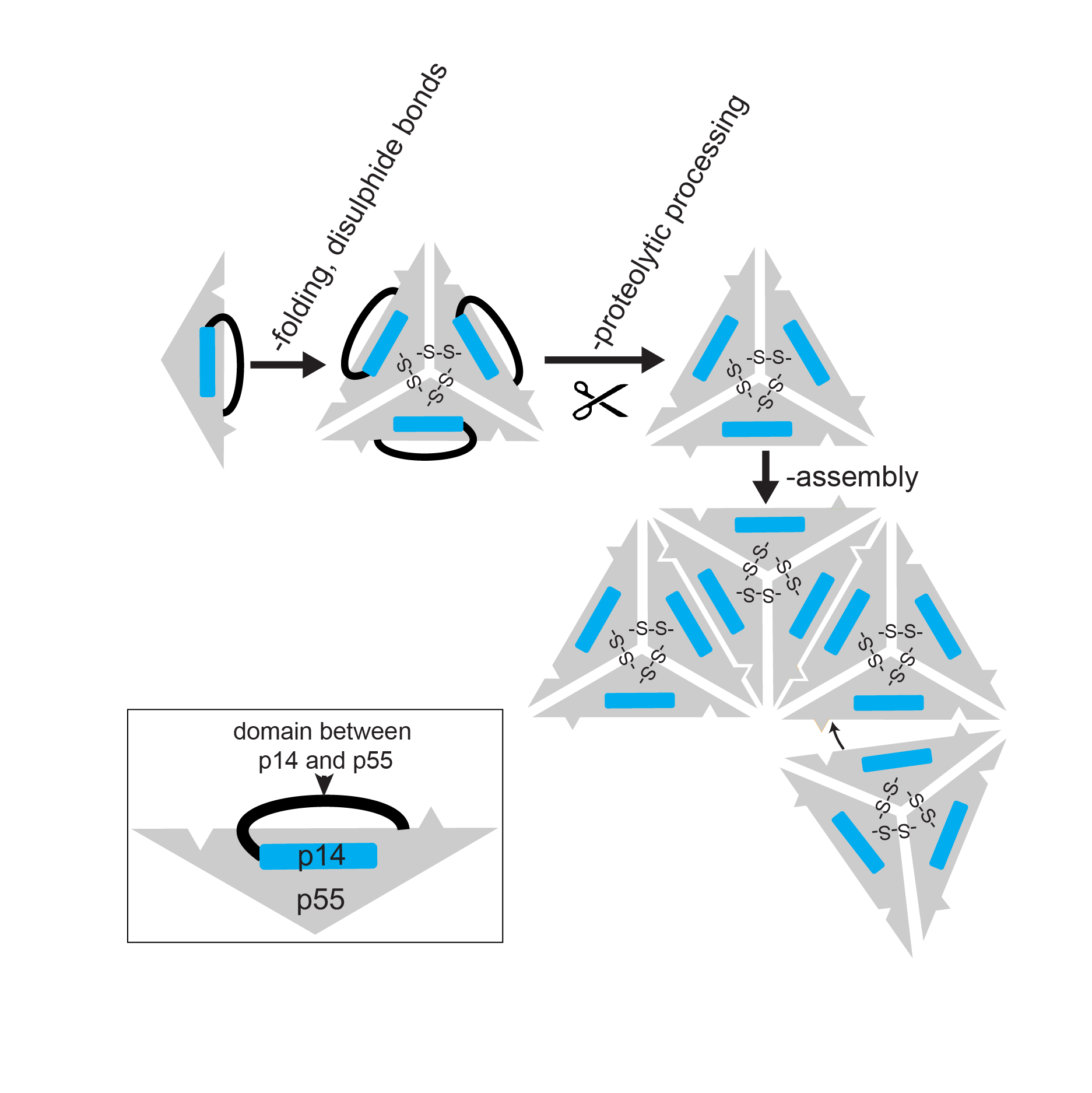

Supplement: S11 Fig — The boxed cartoon depicts different regions of full-length OCTIN. The formation of a 3-dimensional protein lattice requires a minimum of 3 intermolecular contacts. For simplicity, the disulphide crosslinked p55 sub-assembly is depicted as a trimer and the lattice is depicted in two dimensions. We speculate that non-covalent contacts required for assembly are shielded by the region between p14 and p55, which is removed by proteolytic processing. The folding and processing events could occur simultaneously or in the opposite order to that depicted. Note that the role of p14 in lattice assembly remains unclear. OCTIN, octahedral crystal matrix protein. (TIF) [file pbio.2004920.s011.tif]
