# Supplementary material for: Evolutionary novelty in gravity sensing through horizontal gene transfer and high-order protein assembly
Source: PLoS Biol. 2018 Apr 24;16(4):e2004920. doi: 10.1371/journal.pbio.2004920 (PMC5915273; doi:10.1371/journal.pbio.2004920)
Supplement: S1 Text — OCTIN, octahedral crystal matrix protein. (PDF) [file pbio.2004920.s014.pdf]

S1 Text. Biophysical constraints preclude OCTIN's function in bacterial gravitropism.

The hypothesis that OCTIN does not function in bacterial gravitropism is supported in several ways. First, there is no record of gravitropism in bacteria of all shapes and sizes, including filament-forming species capable of developing elaborate aerial structures. Second, Phycomyces OCTIN crystals are octahedrons averaging 5 µm in edge length. The volume of such a crystal is approximately 60 times that of a typical bacterial cell of length 2 µm and diameter 0.5 µm. In bacterial species possessing OCTIN, the cell diameter range of 0.3-0.8 µm [62–67] imposes a corresponding constraint on the size of cytoplasmic bodies. This constraint is amplified within the periplasm, where gram-negative OCTINs are targeted (Fig 2 and 4A).

Using reported values of Phycomyces crystal density (on average, ρ_o = 1.435 g/cm^3, [7]), cytoplasm density (ρ_c = 1.115 g/cm^3, [7]) and cytoplasm viscosity (μ = 0.025 Pa·s, [8]), sedimentation velocity (v) is estimated for hypothetical OCTIN assemblies by Stokes' law, where *r* is the assembly radius:

$$v = \frac{2(\rho_o - \rho_c)gr^2}{9\mu}, [9]$$

The estimated sedimentation velocities of assemblies that can form within the bacterial periplasm or cytoplasm are approximately two to four orders of magnitude lower than that of *Phycomyces* crystals (S10 Fig). Furthermore, the Péclet number (*Pe* = $\frac{4\pi(\rho_o - \rho_c)gr^4}{3k_BT}$ where kB is the Boltzman constant and T is temperature), which describes the ratio of particle movement by gravitational force relative to Brownian motion [10], is less than 0.1 for hypothetical bacterial OCTIN assemblies in this size range. This indicates that Brownian motion dominates the movement of these assemblies, which prevents sedimentation [10]. This result is in agreement with estimations made based on the distribution of particles along a given distance due to the combined effect of terrestrial gravity and Brownian motion [11]. Taken together, these sedimentation properties of hypothetical OCTIN assemblies thus further argue against a function in bacterial gravitropism.

References

1. Mannisto MK, Rawat S, Starovoytov V, Haggblom MM. Granulicella arctica sp. nov., Granulicella mallensis sp. nov., Granulicella tundricola sp. nov. and Granulicella sapmiensis sp. nov., novel acidobacteria from tundra soil. Int J Syst Evol Microbiol. Microbiology Society; 2012;62: 2097–2106. doi:10.1099/ijs.0.031864-0
2. Ward NL, Challacombe JF, Janssen PH, Henrissat B, Coutinho PM, Wu M, et al. Three genomes from the phylum Acidobacteria provide insight into the lifestyles of these microorganisms in soils. Appl Environ Microbiol. 2009;75: 2046–56. doi:10.1128/AEM.02294-08
3. Dworkin M, Falkow S. The prokaryotes. Vol. 5. Proteobacteria : alpha and beta subclasses : a handbook on the biology of bacteria [Internet]. Springer; 2006. Available: https://www.springer.com/us/book/9780387307459
4. García-Fraile P, Benada O, Cajthaml T, Baldrian P, Lladó S. Terracidiphilus gabretensis gen. nov., sp. nov., an Abundant and Active Forest Soil Acidobacterium Important in Organic Matter Transformation. Löffler FE, editor. Appl Environ Microbiol. 2015;82: 560–9. doi:10.1128/AEM.03353-15
5. Rawat SR, Männistö MK, Starovoytov V, Goodwin L, Nolan M, Hauser L, et al. Complete genome sequence of Terriglobus saanensis type strain SP1PR4(T), an Acidobacteria from tundra soil. Stand Genomic Sci. 2012;7: 59–69. doi:10.4056/sigs.3036810
6. Poindexter JS. Asticcacaulis. Bergey’s Manual of Systematics of Archaea and Bacteria. Chichester, UK: John Wiley & Sons, Ltd; 2015. pp. 1–14. doi:10.1002/9781118960608.gbm00790
7. Zalokar M. Intracellular centrifugal separation of organelles in Phycomyces. J Cell Biol. 1969;41: 494–509. doi:10.1083/jcb.41.2.494
8. van den Bogaart G, Hermans N, Krasnikov V, Poolman B. Protein mobility and diffusive barriers in Escherichia coli: consequences of osmotic stress. Mol Microbiol. 2007;64: 858–71. doi:10.1111/j.1365-2958.2007.05705.x
9. Lamb H. Hydrodynamics. 6th ed. Cambridge University Press; 1994.
10. Tanner RI. Rigid particle microstructures. Engineering Rheology. 2nd ed. Oxford University Press; 2002.
11. Galland P. The sporangiophore of Phycomyces blakesleeanus: A tool to investigate fungal gravireception and graviresponses. Plant Biol. 2014;16: 58–68. doi:10.1111/plb.12108
