# Supplementary material for: Evolutionary novelty in gravity sensing through horizontal gene transfer and high-order protein assembly
Source: PLoS Biol. 2018 Apr 24;16(4):e2004920. doi: 10.1371/journal.pbio.2004920 (PMC5915273; doi:10.1371/journal.pbio.2004920)
Supplement: S2 Table — ML, maximum likelihood. (DOCX) [file pbio.2004920.s016.docx]

|  | **Acido/Proteobacteria monophyly constraint** | |
| --- | --- | --- |
|  | + | - |
| **LnL** | -5656.17 | -5595.06 |
| **ΔLnL** | -61.10 | |
| **pAU** | 0.009 | |
| **pSH** | 0.009 | |
| **pKH** | 0.008 | |
| **pRELL** | 0.007 | |
